# Unilateral Degenerative Facet Joint Pathology Eliciting Rapidly Progressive Cervical Spondylotic Myelopathy

**DOI:** 10.7759/cureus.14238

**Published:** 2021-04-01

**Authors:** Yasuhiro Takeshima, Ai Okamoto, Shohei Yokoyama, Ichiro Nakagawa, Hiroyuki Nakase

**Affiliations:** 1 Neurosurgery, Nara Medical University, Kashihara, JPN

**Keywords:** cervical spine, degenerative, facet joint, laminoplasty, myelopathy, rapid deterioration, spondylosis

## Abstract

Cervical spondylotic myelopathy (CSM) is a common age-related disease, but little is known concerning the impact of cervical facet degeneration in patients suffering from CSM without degenerative cervical spondylolisthesis or cervical instability. We report an instructive case of CSM with rapid neurological deterioration in which unilateral degenerative facet joint pathology at the affected spinal level and impressive radiological findings were observed.

A 70-year-old female progressively developed C5 segmental myelopathy without any trauma within a two-week period. Radiological findings revealed C3-4 spinal canal stenosis with the emergence of increased intramedullary signal intensity on T2-weighted magnetic resonance imaging, articular surface damage at the left C3-4 facet joint on computed tomography, and unilateral “facet joint gap” on cervical radiogram, but did not meet the criteria of cervical instability or spondylolisthesis.

This case suggests that some types of severe degenerative changes in cervical facet joints may contribute to cervical myelopathy, especially in cases with progressive neurological deterioration.

## Introduction

Cervical spondylotic myelopathy (CSM) is a common age-related disease that worsens quality of life [1]. Cervical spondylosis that causes CSM generally results from degenerative changes in the intervertebral discs and facet joints [2]. Degenerative cervical facet joint pathology is known to be associated with neck pain and cervical radiculopathy [3]. Moreover, severe degenerative change may indicate degenerative cervical spondylolisthesis (DCS) with accompanying yellow ligament hypertrophy and facet subluxation [4]. However, little is known concerning the impact of cervical facet degeneration in patients suffering from CSM without DCS or cervical instability.

We report an instructive case of CSM with rapid neurological deterioration in which unilateral degenerative facet joint pathology at the affected spinal level and impressive radiological findings were observed.

## Case presentation

**Figure 1:**
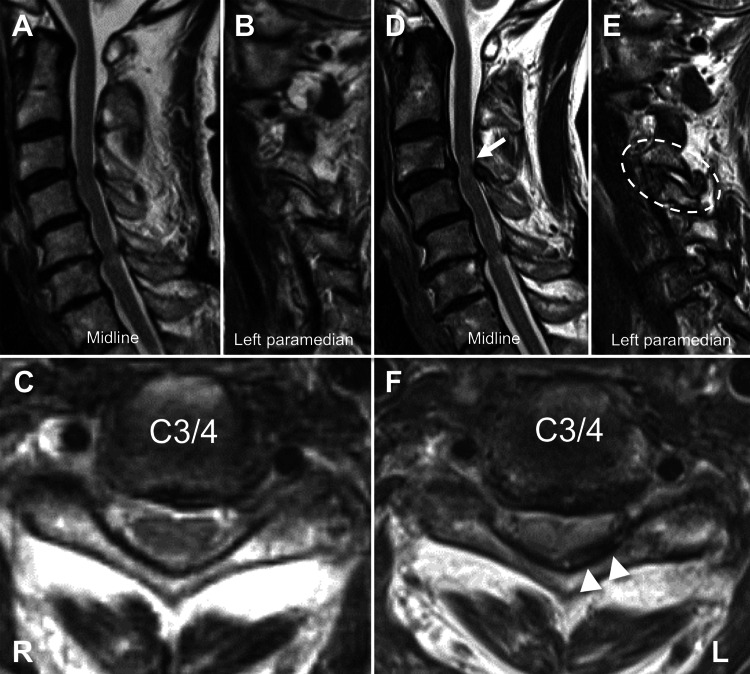
Preoperative T2-weighted magnetic resonance images 15 months (A–C) and just (D–F) before surgery. Sagittal images demonstrate increased intramedullary signal intensity (arrow) and enlargement of the intra-articular gap in the left C3-4 facet joint (circle). Axial images indicate the emergence of asymmetrical ligamentum flavum hypertrophy (arrowheads).

A 70-year-old female had mild bilateral finger numbness due to C5-6 spinal canal stenosis that was diagnosed with cervical magnetic resonance imaging (MRI) (Figures 1A, 1B). The symptomatic spinal canal stenosis had been stable for several years, but she developed right leg weakness followed by numbness of the extremities, bilateral hand clumsiness, and gait difficulty within a two-week period without a definite head or neck injury. Neurological examination revealed tetraparesis in the muscles peripheral to the deltoid, hyperreflexia below biceps brachii, and a positive Romberg test. She did not feel any pain from the neck movement. She was diagnosed with C5 segmental myelopathy. The Japanese Orthopedic Association (JOA) score for cervical compression myelopathy was seven out of 17. T2-weighted MRI (T2WI) revealed developed C3-4 spinal canal stenosis with increased intramedullary signal intensity, indicating an affected spinal segmental level (Figure 1D). Axial T2WI revealed asymmetrical ligamentum flavum hypertrophy at the C3-4 level on the left side (Figures 1C, 1F). Sagittal T2WI indicated hyperintensities in the ipsilateral C3-4 facet joint, suggesting joint hypermobility (Figure 1E).

**Figure 2:**
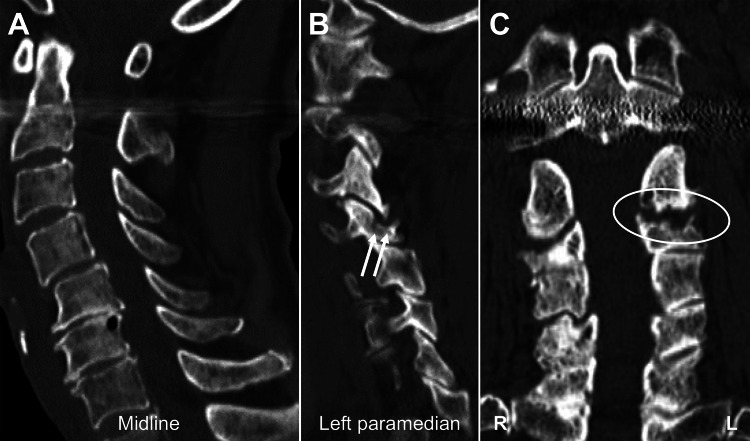
Preoperative cervical computed tomography images. (A) Midline sagittal image demonstrating subaxial spondylotic changes without spinal canal stenosis or spondylolisthesis at the C3-4 level. (B) Left paramedian sagittal image demonstrating a damaged superior articular surface of the left C4 facet joint (arrows). (C) Coronal image demonstrating erosion of the articular surface (circle).

Moreover, computed tomography (CT) showed articular surface damage and erosive changes in the joint without any findings of DCS at the level (Figure 2).

**Figure 3:**
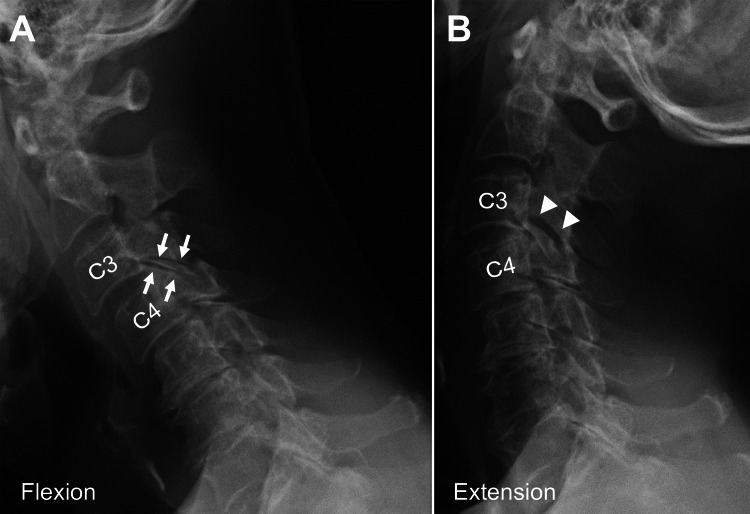
Preoperative lateral dynamic radiographs of the cervical spine. Flexion (A) and extension (B) radiographs reveal C3-4 horizontal translation as 2.5 mm and parallel shadows of the bilateral C3-4 facet joints during flexion (arrows), which enlarge markedly during extension (arrowheads).

Dynamic lateral cervical radiography revealed C3-4 vertebral horizontal translation as 2.5 mm, which did not meet the criteria for cervical instability [5], but one of enlarged parallel shadows during extension suggested the intra-articular gap with laxation in a C3-4 facet joint (Figure 3).

**Figure 4:**
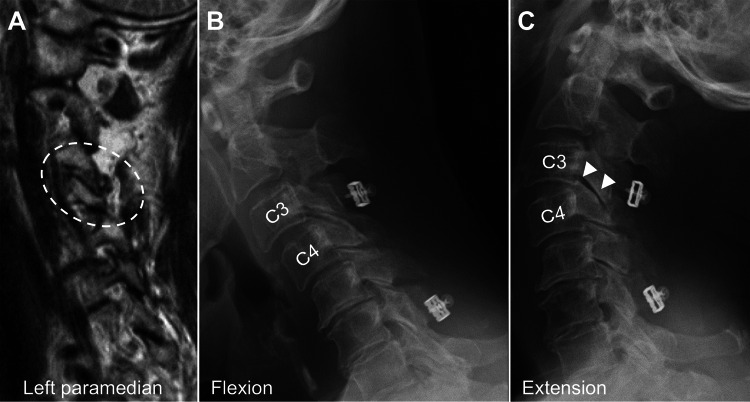
Postoperative cervical radiological images. (A) Left paramedian sagittal T2-weighted magnetic resonance imaging demonstrating the disappearance of interarticular fluid intensities in the C3-4 facet joint (dashed circle). (B, C) Dynamic radiographs showing unchanged C3-4 vertebral body translation and smaller facet joint shadows during extension compared with preoperative images (arrowheads).

Cervical laminoplasty without fusion was performed immediately, and her JOA score improved to 11. She was immobilized with cervical collar for six weeks after the operation. Postoperative sagittal T2WI at six months showed resolution of hyperintensities in the left C3-4 facet joint (Figure 4A). Dynamic lateral radiography 12 months after the surgery at the last follow-up showed unchanged C3-4 vertebral body translation and decrease in the size of shadows on the affected facet joint (Figures 4B, 4C).

## Discussion

Intervertebral discs and facet joints are important for biomechanical shifting of stress on the cervical spine and mobility [3]. Therefore, age-related degeneration, called cervical spondylosis, sometimes indicates clinical issues. It is well known that cervical facet degeneration may indicate not only radiculopathy due to foraminal spinal stenosis [6] but myelopathy in the setting of DCS or cervical instability due to spinal canal stenosis [7]. However, little is known concerning the impact of cervical facet degeneration in patients suffering from CSM without DCS or cervical instability. One reason might be because it is sometimes more difficult to determine the actual affected spinal segment in CSM patients without DCS or cervical instability. It is quite common for patients with symptomatic CSM to have multilevel spinal canal stenosis on radiological examination; false-positive or asymptomatic spinal degeneration is not unusual [8], especially in elderly patients. In the present case, it was fortunately easy to determine the actual affected spinal segment. First, neurological findings clearly indicated C5 myelopathy. Second, because serial radiological imaging was performed for a long time preoperatively, it was possible to detect the true pathological lesion based on temporal and quantitative changes.

In the present case, several impressive radiological findings were observed at the affected spinal level: unilateral facet degeneration with intra-articular T2WI hyperintensities, ipsilateral ligamentum flavum hypertrophy, and a unilateral “facet joint opening” sign. In general, DCS is frequently accompanied by yellow ligament hypertrophy and facet arthropathy and subluxation [4]. Therefore, it is easy to speculate in the present case that hypermobility of the affected cervical facet joint indicates ipsilateral ligamentum flavum hypertrophy and cervical myelopathy. The emergence of increased intramedullary signal intensity on T2WI would support this hypothesis.

Cervical facet joints play key roles in static stability and gliding cervical motion. Therefore, impairment of these joints might negatively impact dynamic cervical motion, ultimately resulting in DCS or presentation of instability at the spinal level [4]. In the present case, without any findings of DCS, dynamic lateral radiography findings did not meet the criteria for cervical instability [5], but a unilateral “facet joint gap” sign was clearly observed. This finding suggests that unilateral facet joint impairment could induce cervical myelopathy due to potential hypermobility of the affected facet joint. Therefore, the “facet joint gap” sign may be a useful indicator of pathological CSM due to facet joint potential laxation. Cervical facet joint has attracted less attention until now in the clinical setting without DCS or instability. In the present case, articular surface damage with erosive changes in the affected cervical facet joint was also observed on CT scan. Therefore, further research of qualitative assessments for cervical facet joint using CT scans may be useful in CSM patients, especially in the situation with rapid neurological deterioration.

For the surgical treatment of CSM, not only decompression of the spinal cord but spinal stabilization should be recommended if dynamic factor was involved as the mechanism of clinical progression. On the other hand, there is a report of open-door laminoplasty for elderly CSM patients, indicating that the range of motion (ROM) of all cervical spines was significantly limited after the operation, and there was no significant difference in the clinical results between the presence and absence of DCS [9]. In the present case, cervical laminoplasty without fusion was conducted because of her lowered activities of daily living and the absence of radiological findings of cervical instability, and because postoperative cervical horizontal dynamic translation of the vertebrae was not aggravated. In addition, postoperative radiological findings revealed resolution of hyperintensities in the left facet joint on T2WI and a decrease in the size of “facet joint opening” sign on cervical radiographs. These results may be supported by the speculation that postoperative limitation of ROM of all cervical spines after open-door laminoplasty for elderly patients might restrict the pathological facet joint hypermobility.

## Conclusions

This illustrative case suggested that cervical facet joint degeneration might be associated with rapidly progressive myelopathy in the absence of marked cervical instability. Dynamic lateral radiography is insufficient for the assessment of cervical facet joint degeneration, but the “facet joint gap” sign may be a potential indicator of pathological CSM.
